# A heterozygous mutation in *NOTCH3* in a Chinese family with CADASIL

**DOI:** 10.3389/fgene.2022.943117

**Published:** 2022-11-29

**Authors:** Juyi Li, Tao Luo, Xiufang Wang, Mengjie Wang, Tao Zheng, Xiao Dang, Aiping Deng, Youzhi Zhang, Sheng Ding, Ping Jing, Lin Zhu

**Affiliations:** ^1^ Department of Pharmacy, The Central Hospital of Wuhan, Tongji Medical College, Huazhong University of Science and Technology, Wuhan, Hubei, China; ^2^ Department of Neurology, The Central Hospital of Wuhan, Tongji Medical College, Huazhong University of Science and Technology, Wuhan, Hubei, China; ^3^ Department of Pain, The Central Hospital of Wuhan, Tongji Medical College, Huazhong University of Science and Technology, Wuhan, Hubei, China; ^4^ Department of Endocrinology, The Central Hospital of Wuhan, Tongji Medical College, Huazhong University of Science and Technology, Wuhan, Hubei, China; ^5^ Department of Pharmacy, Taihe Hospital, Hubei University of Medicine, Shiyan, Hubei, China; ^6^ Department of Prenatal Diagnostic Center, Guangzhou Women and Children’s Medical Center, Guangzhou Medical University, Guangdong, China; ^7^ School of Pharmacy, Hubei University of Science and Technology, Xianning, China; ^8^ Department of Pediatrics, Tongji Hospital, Huazhong University of Science and Technology, Wuhan, Hubei, China

**Keywords:** CADASIL, whole-exome-sequencing, heterozygous, Notch3, treatment scheme

## Abstract

**Introduction:** Cerebral autosomal-dominant arteriopathy with subcortical infarcts and leukoencephalopathy (CADASIL) is an autosomal-dominant systemic vascular disease that primarily involves small arteries. Patients with CADASIL experience migraines, recurrent ischemic strokes, cognitive decline, and dementia. The *NOTCH3* gene, which is located on chromosome 19p13.12, is one of the disease-causing genes in CADASIL. Herein, we investigate the genetic and phenotypic features in a Chinese CADASIL family with heterozygous *NOTCH3* mutation.

**Methods and Results:** In the family, the proband suffered from dizziness, stroke, and cognitive deficits. Brain magnetic resonance imaging (MRI) demonstrated symmetrical white matter lesions in the temporal lobe, outer capsule, lateral ventricle, and deep brain. Whole-exome sequencing identified a known missense mutation in the proband, c.397C>T (p.Arg133Cys), which was identified in his son and granddaughter using Sanger sequencing. The proband’s younger brother and younger sister also have a history of cognitive impairment or cerebral infarction, but do not have this genetic mutation, which may highlight the impact of lifestyle on this neurological disease.

**Conclusion:** We identified a known CADASIL-causing mutation *NOTCH3* (c.397C>T, p.Arg133Cys) in a Chinese family. The clinical manifestations of mutation carriers in this family are highly heterogeneous, which is likely a common feature for the etiology of different mutations in CADASIL. Molecular genetic analyses are critical for accurate diagnosis, as well as the provision of genetic counselling for CADASIL.

## Introduction

Cerebral autosomal-dominant arteriopathy with subcortical infarcts and leukoencephalopathy (CADASIL) is a rare autosomal-dominant, inherited, small-cerebral vessel disease which is characterized by migraines, recurrent stroke, mood disturbances, apathy, dementia, and premature death (Kalimo et al., 2002; Tikka et al., 2014). There are two types of CADASIL, CADASIL1 and CADASIL2, according to gene mutations: notch receptor 3 gene (*NOTCH3*) and HtrA serine peptidase 1 gene (*HTRA1*), respectively (Joutel et al., 1996; Verdura et al., 2015). The *NOTCH3* mutation is the predominant genetic cause of CADASIL, and at least 230 *NOTCH3* mutations are known and can be found in the Human Gene Mutation Database (http://www.hgmd.cf.ac.uk/). NOTCH3 is a single-pass transmembrane receptor that is required for vascular smooth muscle cell (VSMC) differentiation and maturation, and the integrity of especially resistance arterioles (Henshall et al., 2015). The NOTCH3 extracellular domain (NOTCH3^ECD^) contains 34 epidermal growth factor-like repeat (EGFr) domains, which are ∼40 amino acid-long modular protein subunits, characterized by a conserved number and position of six cysteine residues. In pairs, these cysteines form three disulphide bridges that stabilize the EGFr domain structure (Joutel et al., 1996). Most *NOTCH3* mutations that cause CADASIL change the number of cysteine residues in the EGF-like structure, which causes an abnormal accumulation of aberrant NOTCH3 molecules in the basement membrane of the vascular smooth muscle cells. The *NOTCH3* mutation causes CADASIL, not because it affects the normal functioning of NOTCH3, but because it increases a toxic effect that was not present before (Domenga et al., 2004).

Recent studies have demonstrated that the incidence of these pathogenic mutations in the population is as high as 1:300 (Rutten et al., 2016), much higher than what was previously reported, suggesting that a considerable number of patients or asymptomatic carriers are likely misdiagnosed. The high heterogeneity of phenotypes among individuals with CADASIL increases the difficulty of disease diagnosis and management. Therefore, a follow-up analysis of the clinical symptoms and the natural course of CADASIL patients can help explore the pathogenesis of CADASIL and aid in individual management. The aim of this study was to identify gene mutations-of-interest in a Han Chinese patient with clinical presentations strongly suggestive of CADASIL.

## Materials and methods

### Patient and clinical data

The study was granted approval by the Wuhan Central Hospital ethics committee, approval number [2020-189]. Informed consent was acquired from the proband’s family. The clinical data were collected through interviews, physical examination, brain magnetic resonance imaging (MRI), and medical history and reports.

### Sample preparation and whole-exome sequencing

Genomic DNA from the proband’s peripheral blood samples were extracted by using a DNA extraction kit (TIANGEN, Beijing, China). Subsequently, next-generation sequencing of the proband’s DNA was conducted, according to the SureSelect Human All Exon V5 kit (Agilent) (Zenagui et al., 2018). Additionally, exome sequencing was processed in the HiSeq2500 system (Illumina).

### Genetic analyses

The sequencing reads were pre-processed in order to reduce the noise, and clean data were mapped and aligned to the Human Reference Genome (UCSC hg19, NCBI build 37). Duplicated sequencing reads were removed using Picard (http://broadinstitute.github.io/picard/). Realignment was conducted using the Genome Analysis Toolkit (http://www.broadinstitute.org/gatk/guide/best-practices). Variants including SNVs and indels were referenced to the public databases (1000 Genomes, ESP, dbSNP, ExAC, and gnomAD). Variants in the genes that are involved in CADASIL, published by PubMed and OMIM, were analyzed. The non-synonymous coding variants, frameshift indel variants, and splice-site variants were also analyzed. On the other hand, non-exonic and synonymous variants were excluded. The functional impact of missense variation was evaluated using silico prediction tools, including SIFT, PolyPhen2, Mutation Taster, Mutation Assessor, GERP-plus, PhyloP100, and PhastCons100 (Iacovazzo et al., 2018; Pace et al., 2018; Li et al., 2020).

### Sanger sequencing

Sanger sequencing was performed to validate and co-segregate candidate variants in the proband and his family members. The primers used in PCR amplification for Sanger sequencing were as follows: Forward: 3′-CAG​TCG​TAA​GTG​AGG​TCG​CC-5′, Reverse: 3′- TGC​CCA​ACC​AAG​CCA​TCT-5′. Furthermore, the sequencing primer was 3′- CCC​GTG​GCT​TCC​GAG​GTG-5’.

### Structure modeling

The Swiss–Prot repository was used to simulate a three-dimensional (3D) structure of the NOTCH3 protein with a missense mutation.

## Results

### Patient history and clinical features

After 2006, the proband, 55 years old, started suffering from personality changes over the following 13 years. In particular, the patient became irritable, obsessive, slow responding, had a memory decline (near-memory mainly), and computational power decline. However, he did not experience any auditory or visual hallucinations. In 2014, at the age of 63, the proband was initially admitted to the Wuhan Central Hospital for “sudden psychotic symptoms.” In the following 5 years, he was hospitalized 10 times with recurrent dizziness and mental illness. Furthermore, he had a medical history of kidney stones, gastric ulcers, left facial neuritis, and hyperuricemia. The patient did not present with any cerebrovascular risk factors including hypertension, high glucose, hypercholesterolemia, and smoking.

**FIGURE 1:**
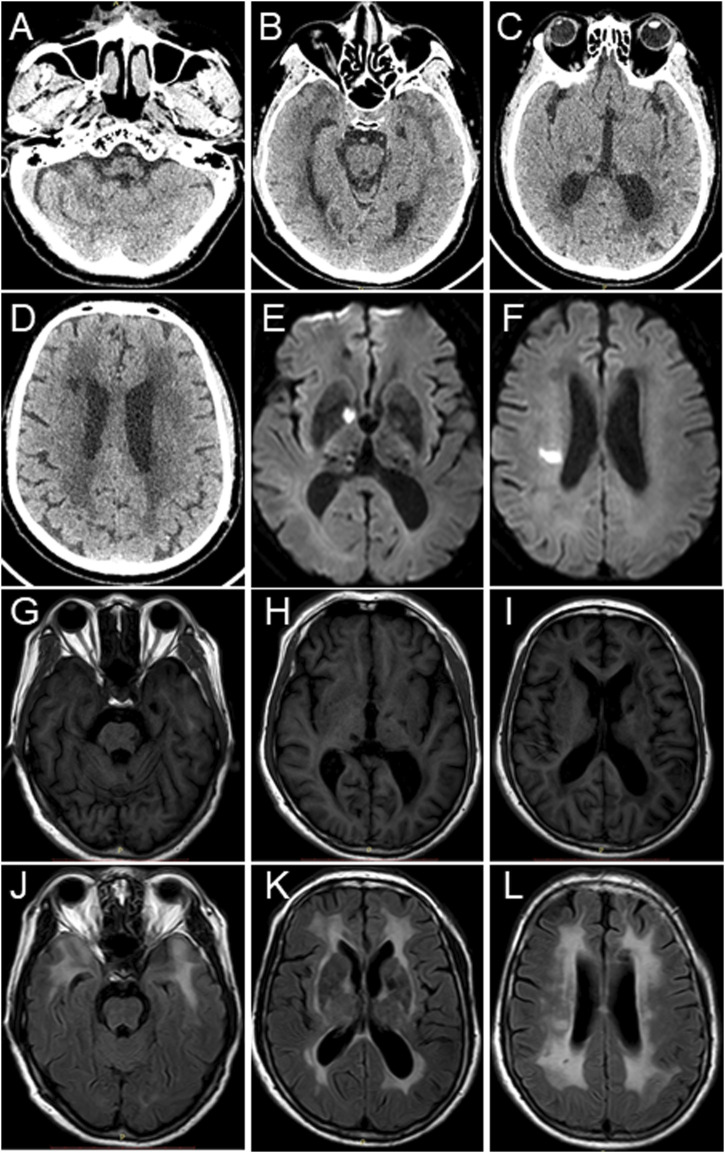
Brain CT scan and MRI images of the proband. **(A–D)** Brain CT scan suggests multiple lacunar cerebral infarctions, white matter degeneration, and brain atrophy. **(E,F)** Brain MRI-DWI shows significant hyperintensity signals. **(G–I)** Decreased signal intensities were observed in the brain stem, basal ganglia, and thalamus in the T1-weighted MRI images. **(J–L)** T2-weighted and fluid-attenuating inversion recovery (FLAIR) brain MRI revealed symmetrical white matter lesions in the temporal pole, outer capsule, lateral ventricle, and deep brain.

**FIGURE 2:**
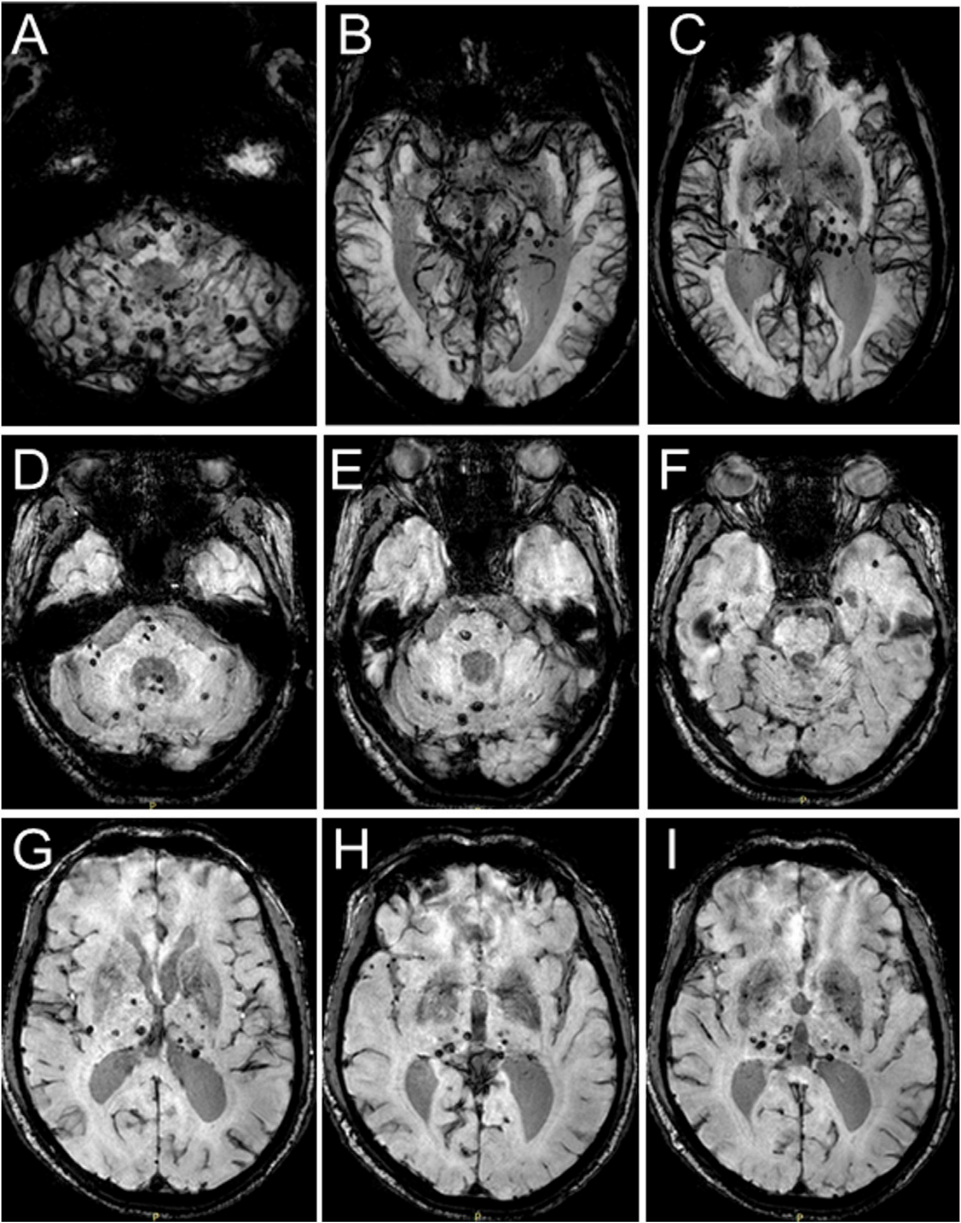
Brain MRI-SWI images of the proband. SWI images present with multiple hypointense signal intensities at several brain levels and demonstrate multiple foci of hemorrhage in the proband’s brain.

**FIGURE 3:**
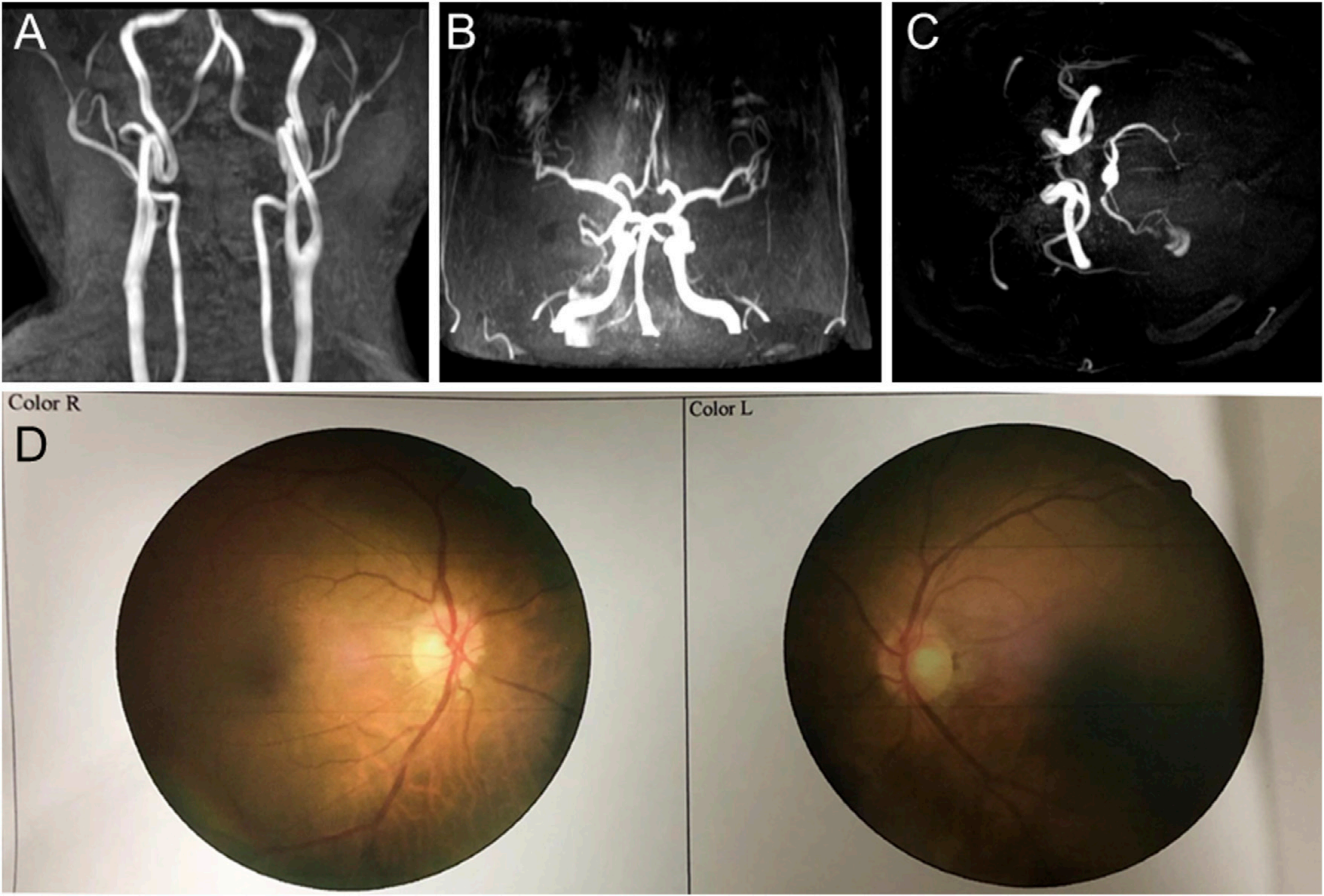
Craniocerebral DSA (digital subtraction angiography) and fundus arteriography images of the proband. **(A–C)** DSA images reveal multiple stenoses of the cerebral artery. **(D)** Fundus arteriography images showed a part of the branch copper wire sign.

In 2018, the proband (67 years of age) suffered his first acute ischemic attack, an acute cerebral infarction at the left edge of the brainstem. Then, 10 months later (May 2019), he developed multiple small intracranial hemorrhages. Subsequently, in September 2019 (the 11th hospitalization), he experienced an acute ischemic attack again, an acute lacunar infarction in the right basal ganglia and radio-corona. The main symptom was weakness in both legs, accompanied by transient reaction dullness, difficulty in speech expression, speech disorder, and partial impairment of understanding. Results from a brain CT scan suggested multiple lacunar cerebral infarctions, white matter degeneration, and brain atrophy (Figures 1A–D). Significant high-density signals were observed in the brain DWI (diffusion weighted imaging), suggesting acute ischemic lesions in the right basal ganglia region and radio-corona region (Figures 1E, F). The brain stem, basal ganglia, and multiple luminal infarcts in the thalamus could be readily observed on the T1-weighted imaging with decreased signal intensity (Figures 1G–I). T2-weighted and FLAIR (fluid-attenuating inversion recovery) brain MRI revealed symmetrical white-matter lesions in the temporal pole, outer capsule, lateral ventricle, and deep brain (Figures 1J–L). Additionally, the SWI (susceptibility weighted imaging) revealed multiple low signals, which were thought to be micro-bleeding (Figures 2A–I). His craniocerebral DSA (digital subtraction angiography) examination revealed multiple things. First, a slight stenosis of the left internal carotid artery in the siphon segment was found, as well as multiple moderate-to-severe stenosis of the right anterior-cerebral artery and multiple mild-to-moderate stenosis of the left-middle cerebral artery. Second, multiple mild-to-moderate bilateral-posterior cerebral artery stenoses were identified, as well as a bilateral-superior cerebellar artery with multiple moderate stenosis (Figures 3A–C).

**FIGURE 4:**
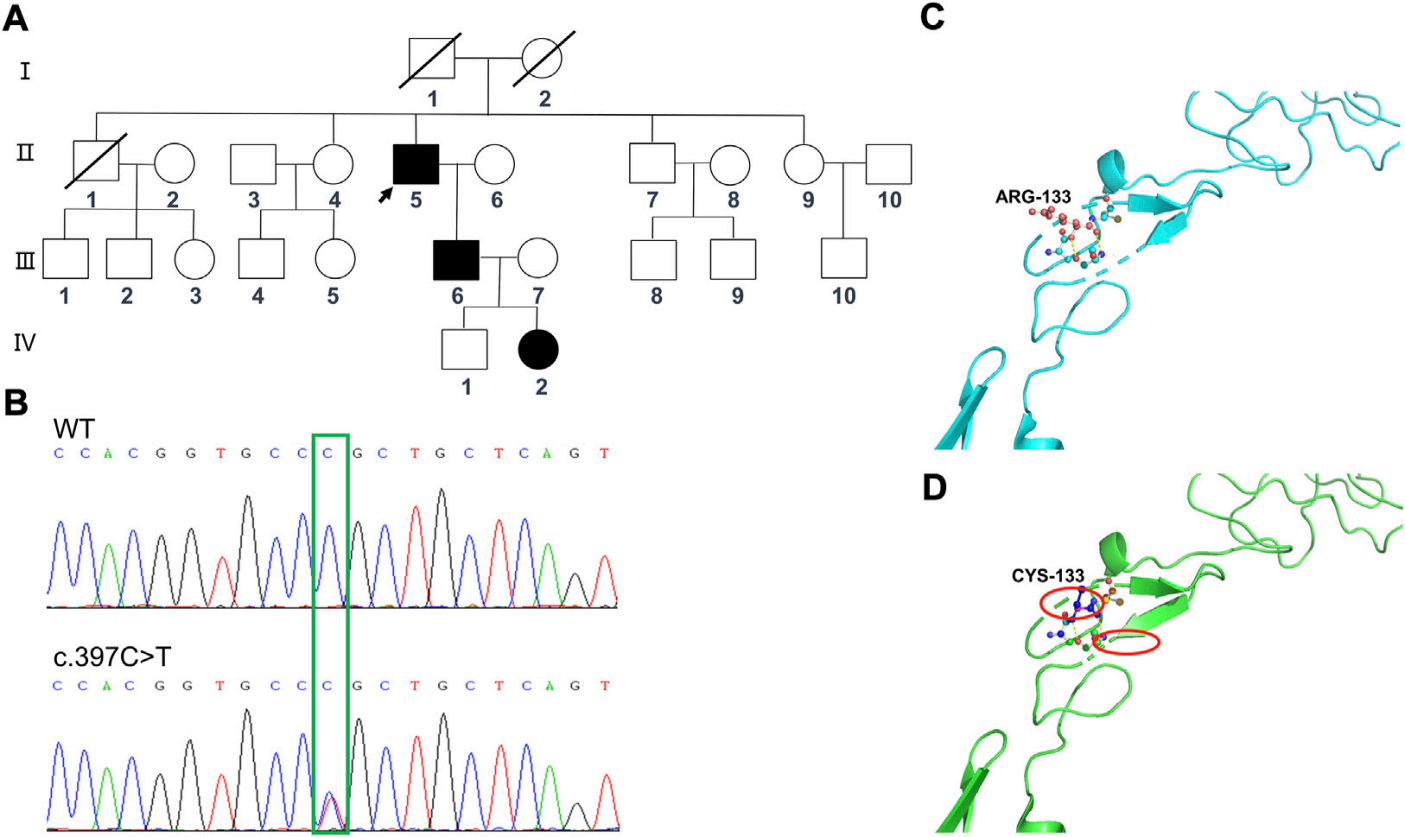
Sequencing analysis of the NOTCH3 (c.397C>T) mutations in the family members, and predicted computational tertiary structure of the NOTCH3 protein. **(A)** Squares denote male family members; circles denote female family members; slashed symbols represent deceased family members; fully shaded symbols represent symptomatic family members; and arrows indicate the proband. **(B)** The affected nucleotide is indicated with an arrowhead. The family members Ⅱ:6, Ⅱ:7, Ⅱ:9, Ⅲ:8, Ⅲ:9, Ⅲ:10, and Ⅳ:1 are wildtypes, while the family members Ⅱ:5, Ⅲ:6, and Ⅳ:2 have the c.397C>T mutation. The predicted computational tertiary structure of the wild genotype NOTCH protein **(C)** and the mutant genotype **(D)**. An amino acid change resulting from a non-synonymous substitution, with arginine replacing cysteine.

Based on clinical presentations and neuroimaging findings, the patient was clinically suspected of having CADASIL. We traced the patient’s medical and family history (Figure 4A) and discovered that his mother had a history of cerebrovascular disease. Additionally, his old brother died at 65 years of age with a cerebral infarction and a suspected history of dementia. His old sister, who was 70 years old (2020), still alive, had memory problems and a “weird” personality. At the age of 66 (2017), the proband was diagnosed with retinal arteriosclerosis. Fundus arteriography images (Figure 3D) revealed a part of the branch copper wire sign (early arterial narrowing, thickening of the wall, and enhanced reflection, like a copper wire), which is a typical clinical manifestation of CADASIL involving the fundus artery. Then, the patient and some of his family members underwent genetic and pathological analyses.

### Mutation screening and characteristics of family members

Whole-exome sequencing was performed for the proband (Ⅱ:5) (Table 1), from which a total of 110,581 SNPs and 11,581 InDels were detected. Variants were filtered according to their frequency, location, and functional consequences. Thus, we were able to identify a heterozygous mutation (c.397C>T, p.Arg133Cys) in exon 4 of the *NOTCH3* gene (Figure 4B), which is known to be a pathogenic mutation (rs137852642) (Yin et al., 2009). Furthermore, the same heterozygous mutation was detected in his son (Ⅲ:6), a 41-year-old man who had become emotional over the last 3 years, and his granddaughter (Ⅳ:2), an 11-year-old girl with no symptoms or signs of CADASIL to date. We have not detected any potentially harmful pathogenic mutations in the *HTRA1* gene.

**TABLE 1:** Whole-exome sequencing detail

Exome capture statistics	Proband
Target region (bp)	60,456,963
Clean reads (bp)	116,114,648
Clean bases (Mb)	11,611,464,800
Mapped reads (bp)	115,726,751
Mapped bases (Mb)	11,550,057,192
Mapping rate (%)	99.67
Reads mapped to target region (bp)	54,986,140
Capture specificity (%)	47.51
Duplication rate (%)	13.87
Uniq rate(%)	98.11
Bases mapped to target region (Mb)	4,432,073,165
Mean depth of target region (X)	73.31
Coverage of target region (%)	97.2
Fraction of target covered ≥4X (%)	94.28
Fraction of target covered ≥10X (%)	90.27
Fraction of target covered ≥20X (%)	82.36
Fraction of target covered ≥30X (%)	72.7
Fraction of target covered ≥50X (%)	52.86
Bases mapped to flanking region (Mb)	2,234,188,443
Mean depth of flanking region (X)	47.57
Coverage of flanking region (%)	91.7
Fraction of flanking covered ≥4X (%)	85.58
Gender	Male

Family member I:2, the proband’s mother, suffered from dizziness and cerebral hemorrhage when she was 40 years of age, which resulted in hemiplegia and death at 65 years. The family member Ⅱ:1, the proband’s elder brother, who suffered from cerebral infarction at 50 years of age, was diagnosed with a cerebrovascular disease and died at 65 years of age. Family member Ⅱ:4, the proband’s elder sister, who was 70 years old, suffered from cerebral infarction and is now currently being treated with oral aspirin. There were no neurological examinations done on these three family members, though their symptoms and signs are consistent with CADASIL. Unfortunately, we were not able to obtain DNA samples from the three and did not carry out relevant genetic tests. Thus, we could not determine whether they had CADASIL or not.

Family member Ⅱ:7, the proband’s younger brother, 64 years old, and suffered from blurry vision and a poor sense of direction. Family member Ⅱ:9, the proband’s younger sister, 61 years old, developed symptoms of daze and suffered from mild cerebral infarction at the age of 54. Then, she was treated with oral aspirin and salviae miltiorrhiza (a kind of Chinese patent medicine). At present, her symptoms of daze improved, but symptoms of hand and foot numbness have appeared. However, genetic tests on both showed no disease-causing mutations (*NOTCH3*, c.397C>T, p.Arg133Cys).

### Protein structure prediction

The tertiary protein structure homology models of the wild-type and p.Arg133Cys NOTCH3 proteins were predicted by Swiss–Prot (Figures 4C, D). We compared the mutated protein structure to the normal protein structure. An amino acid change resulted from a non-synonymous substitution, in which an arginine was replaced by cysteine. A close-up of an amino acid change analysis demonstrated that the number of cysteine residues in the EGF-like structure was no longer even, which likely caused an improper oligomerization and change in protein function.

## Discussion and conclusion

Herein, we described the clinical features and *NOTCH3* gene mutations in one Chinese CADASIL family. The clinical features of the proband include recurrent dizziness, stroke, and cognitive deficits. The confluent white matter hyperintensities, as well as multiple lacunar infarcts, shown during the brain MRI scanning of the proband were in accordance with the typical neuroimaging features of CADASIL (Davous, 1998). A diagnosis of CADASIL was confirmed by *NOTCH3* gene mutations, and one previously reported mutation, c.397C>T, was identified in our study.

The mutation was located on chromosome 19, position15303053, c.397C>T, NM_000435.3, and p.Arg133Cys (rs137852642). An analysis of the evolutionary conservation was conducted on the p.Arg133Cys mutation. The data indicate that this mutation is highly conserved among multiple animal species (Table 2). As a result of this mutation, the number of cysteine residues in the EGF-like structure is no longer even, so there may be at least two pathogenic mechanisms. First, a change in the number of cysteine residues in the EGF-like structure affects the normal three-dimensional configuration of the NOTCH3 molecule, which affects its function or increases the previously unknown toxic effect. Second, unpaired cysteine residues may form peptide bonds with other NOTCH3 molecules, or other proteins, containing unpaired cysteine residues, which may lead to improper oligomerization (Dichgans et al., 2000). Further analysis showed that Notch3^ECD^ accumulation promotes the sequestration of multiple proteins from the microvascular extracellular matrix such as tissue inhibitor of metalloproteinases 3 (TIMP3) and vitronectin (Monet-Leprêtre et al., 2013). Excess TIMP3, which forms complexes with Notch3^ECD^, and abnormally accumulates in the extracellular matrix of mutant brain vessels, and blunts the activity of the ADAM17/HB-EGF/(ErbB1/ErbB4) pathway, thereby attenuating myogenic responses in the brain’s arteries and compromises cerebral blood flow (CBF) regulation in CADASIL. However, the exact pathogenic mechanism of the NOTCH3 mutation in our study is an important content for our future research.

**TABLE 2:** Evolutionary conservation analysis for the p.Arg133Cys mutation in NOTCH3.

Protein acc.	Gene	Organism	Amino acid sequences		
NP_000426.2	NOTCH3	H. sapiens	128	CAHGARCSVGPDGRFLCSCPPGYQGRSCRSDVDECRVGEPCRHGGTCLNT	177
XP_003316212.1	NOTCH3	P. troglodytes	128	CAHGARCSVGPDGRFLCSCPPGYQGRSCRSDVDECRVGEPCRHGGTCLNT	177
XP_002808222.1	NOTCH3	M. mulatta	352	CAHSARCSVGPDGRFLCSCPPGYQGRSCRSDVDECRVGEPCRHGGTCLNT	401
XP_005633258.1	NOTCH3	C. lupus	164	CTPWARCSXGPDGRYICSCP------------------------------	183
XP_003586294.1	NOTCH3	B. taurus	129	CAHGARCSVGSDGRYLCSCPPGYQGRSCRSDVDECRMGGPCRHGGTCLNT	178
NP_032742.1	NOTCH3	M. musculus	129	CVHGAPCSVGPDGRFACACPPGYQGQSCQSDIDECRSGTTCRHGGTCLNT	178
NP_064472.2	NOTCH3	R. norvegicus	130	CAHGAPCSVGSDGRYACACPPGYQGRNCRSDIDECRAGASCRHGGTCINT	179
XP_002941531.2	NOTCH3	X. tropicalis	113	CENGARCT-NWNGRYNCTCPPGYQGRSCRVDIDECRTPGLCQNGGQCVNT	161

In addition, the proband’s son, a heterozygous carrier of the NOTCH3 c.397C>T (p.Arg133Cys) mutation, had become emotional over the last three years, which is a sign of CADASIL mood disorder. The proband’s granddaughter, another heterozygous carrier in this family, did not have any typical CADASIL symptoms or signs, likely due to her young age or the extremely broad phenotypic spectrum of the NOTCH3 cysteine-altering variants, which ranged from CADASIL to non-penetrance (Rutten et al., 2020). Although clinical and genetic assessments were unavailable from the proband’s parents, we believe that his mother was a likely heterozygous carrier of the *NOTCH3* c.397C>T (p.Arg133Cys) mutation due to her typical symptoms.

Studies have revealed that the identical *NOTCH3* gene mutation often resulted in considerable phenotypic variability in unrelated patients, and even in members of the same family, which suggests additional genetic, environmental, or other vascular risk factors that influence disease progression (Soong et al., 2013). The proband’s younger brother and younger sister have a history of cognitive impairment or cerebral infarction, but do not have the gene mutation (*NOTCH3*, c.397C>T, p.Arg133Cys), which is likely related to their similar lifestyles and other genetic factors with the proband.

CADASIL is characterized by pathological changes in the systematic vasculature, particularly the cerebral arterioles (Donahue and Kosik, 2004; Chabriat et al., 2009; Tang et al., 2017). Identification of the causative mutation is considered to be the gold standard for diagnosing CADASIL (Ishiko et al., 2006). Mutations in *NOTCH3*, which maps to chromosome 19p13.12, are the genetic causes for CADASIL (Tikka et al., 2014; Verdura et al., 2015; Maksemous et al., 2016). The NOTCH3 protein, a type I transmembrane receptor protein, is primarily expressed in vascular smooth muscle cells (VSMCs) and pericytes (Maksemous et al., 2016; Burkett and Dougherty, 2017; Muiño et al., 2017). In the N-terminus of the NOTCH3 protein, an extracellular domain (NOTCH3^ECD^) consists of 34 epidermal growth factor-like repeats (EGFr) that are encoded by exons 2–24 (Burkett and Dougherty, 2017; Muiño et al., 2017). EGFr 2–5, which are encoded by exons 3 and 4, are the most frequently mutations causing CADASIL (Tikka et al., 2014). The proband, his son, and his granddaughter have the heterozygous mutation (*NOTCH3*, c.397C>T, p.Arg133Cys) in exon 4. The NOTCH3^ECD^ cascade hypothesis, composed of a series of negative events, is considered to be the main pathomechanism associated with CADASIL (Rutten et al., 2016). As the disease progresses, VSMCs degenerate and become granular osmiophilic material (GOM), which refers to the aggregated NOTCH3^ECD^ which is the major component of GOM depositions and accumulates between VSMCs. GOM is unique to CADASIL, but we did not obtain any evidence of GOM deposition of the proband in our study.

Joutel et al. found this specific *NOTCH3* mutation (c.397C>T, p.Arg133Cys) in a Caucasian patient in 1997 (Joutel et al., 1997). Ueda et al. reported that this *NOTCH3* mutation was most frequently reported in Japanese CADASIL patients and significantly more CADASIL patients with this mutation had hyperintensity in the external capsule, compared to CADASIL patients with other mutations, not including the NOTCH3 Arg75Pro mutation (Ueda et al., 2015). Among Finnish CADASIL families, this mutation was also the most frequently detected (18 out of 21 families), and the researchers observed a similar haplotype linked to this mutation among all of the Finnish pedigrees (Mykkänen et al., 2004). However, this specific mutation is rarely reported among the Chinese population (Yin et al., 2009). Thus, more case reports are needed on this mutation in the future so that we are able to better summarize the clinical characteristics of CADASIL induced by this mutation among the Chinese.

The proband had a 55 year age-of-onset and recurrent CADASIL symptoms, including dizziness, stroke, and cognitive deficits. The patient was admitted to the hospital 11 times (2014-2019) before he was diagnosed with CADASIL. The cost of time and money has proven to be overwhelming, but now, there is no therapy available to fundamentally mitigate or prevent the onset of symptoms (Joutel, 2011). However, it is an important strategy to delay or prevent the occurrence of cerebral infarction by preventing the risk factors of vascular diseases.

Nevertheless, treatment of CADASIL still remains the focus of the scientists’ efforts. According to pathogenesis of CADASIL, exclusion of the mutant EGFr domain from NOTCH3 likely eliminates the detrimental effect of unpaired cysteine, thus preventing toxic NOTCH3 accumulation and negative cascading events leading to CADASIL. Researchers introduced a novel approach of cysteine-corrective *NOTCH3* exon skipping using antisense oligonucleotides or CRISPR/Cas9-mediated gene editing. Researchers showed that targeted-*NOTCH3* exon skipping is technically feasible and that NOTCH3 skip proteins undergo normal processing and retain the ligand-binding capacity, as well as ligand-induced activation in CADASIL patient-derived cerebral vascular smooth muscle cells (Rutten et al., 2016) or HEK293 cells (Gravesteijn et al., 2020). Moreover, the key feature of CADASIL pathogenesis is the accumulation of Notch3^ECD^ in small vessels. Thus, immunotherapy targeting Notch3^ECD^, such as the systemic administration of Notch3^ECD^ monoclonal antibodies, may be a new avenue for disease-modifying treatment in CADASIL (Ghezali et al., 2018). Machuca-Parra found that the systemic administration of NOTCH3 antibody agonists prevents mural cell loss, which encompasses pericytes and vascular smooth muscle cells, is a hallmark of CADASIL, resulting in vascular instability, and modifies plasma proteins associated with NOTCH3 activity (Machuca-Parra et al., 2017). This finding opens the door for the treatment of CADASIL by modulating NOTCH3 signaling. Of course, many reagents that are able to improve acute or chronic stroke, such as stem cell factor (SCF) and granulocyte colony-stimulating factor (GCSF) (Liu et al., 2015), can also improve the cognitive functioning of CADASIL patients through its neuroprotection and brain repair effects. Those results appear to be promising, but immense efforts are still needed to validate for translating preclinical results to humans.

In summary, a known CADASIL-causing mutation, *NOTCH3* (c.397C>T, p.Arg133Cys), was identified in our study. The clinical manifestations of the mutation carriers in this family are highly heterogeneous, and this may be a common feature for etiology of different mutations in CADASIL. For patients that are clinically suspected as having CADASIL, exome-sequencing has proven to be a time-saving and cost-effective way to identify causative mutations, which is decisive in the diagnosis of CADASIL and facilitates genetic counseling and treatment. Our study shows that a detailed family history is particularly important in the diagnosis of CADASIL. With the exception of immunotherapy and the exon-skipping strategy, more effective and feasible treatment schemes for CADASIL need to be further explored. With the development of CRISPR/Cas9 gene-editing technology, its application in single-gene or single-base mutated diseases is becoming more and more profound, making it valuable for the treatment of CADASIL.

## Data Availability

The original contributions presented in the study are included in the article/Supplementary Material; further inquiries can be directed to the corresponding authors.

## Abbreviations

CADASIL, cerebral autosomal-dominant arteriopathy with subcortical infarcts and leukoencephalopathy; NOTCH3, Notch receptor 3 gene; HTRA1, HtrA serine peptidase 1 gene; FLAIR, fluid-attenuating inversion recovery; VSMCs, vascular smooth muscle cells; EGFr, epidermal growth factor-like repeats; GOM, granular osmiophilic material; GCSF, granulocyte colony-stimulating factor.
