# Corrigendum: A heterozygous mutation in *NOTCH3* in a Chinese family with CADASIL

**DOI:** 10.3389/fgene.2023.1273023

**Published:** 2023-08-15

**Authors:** Juyi Li, Tao Luo, Xiufang Wang, Mengjie Wang, Tao Zheng, Xiao Dang, Aiping Deng, Youzhi Zhang, Sheng Ding, Ping Jing, Lin Zhu

**Affiliations:** ^1^ Department of Pharmacy, The Central Hospital of Wuhan, Tongji Medical College, Huazhong University of Science and Technology, Wuhan, Hubei, China; ^2^ Department of Neurology, The Central Hospital of Wuhan, Tongji Medical College, Huazhong University of Science and Technology, Wuhan, Hubei, China; ^3^ Department of Pain, The Central Hospital of Wuhan, Tongji Medical College, Huazhong University of Science and Technology, Wuhan, Hubei, China; ^4^ Department of Endocrinology, The Central Hospital of Wuhan, Tongji Medical College, Huazhong University of Science and Technology, Wuhan, Hubei, China; ^5^ Department of Pharmacy, Taihe Hospital, Hubei University of Medicine, Shiyan, Hubei, China; ^6^ Department of Prenatal Diagnostic Center, Guangzhou Women and Children’s Medical Center, Guangzhou Medical University, Guangdong, China; ^7^ School of Pharmacy, Hubei University of Science and Technology, Xianning, China; ^8^ Department of Pediatrics, Tongji Hospital, Huazhong University of Science and Technology, Wuhan, Hubei, China

**Keywords:** CADASIL, whole-exome-sequencing, heterozygous, Notch3, treatment scheme

In the published article, there was an error in the **Funding** statement. “A fund (Central Guiding Local Science and Technology Development Special Project, No. 2022BGE272) which supports our work was not included in the article.” The correct **Funding** statement appears below.

“This study was supported by the Health and Family Planning Commission of Wuhan City (WX18M02) and Central Guiding Local Science and Technology Development Special Project (No. 2022BGE272).”

The authors apologize for this error and state that this does not change the scientific conclusions of the article in any way. The original article has been updated.

